# Energy Sustainability with a Focus on Environmental Perspectives

**DOI:** 10.1007/s41748-021-00217-6

**Published:** 2021-04-22

**Authors:** Marc A. Rosen

**Affiliations:** Faculty of Engineering and Applied Science, University of Ontario Institute of Technology, 2000 Simcoe Street North, Oshawa, ON L1G 0C5 Canada

**Keywords:** Energy sustainability, Energy, Sustainability, Sustainable development, Environment, Ecology, Economics, Society, Equity, Resources

## Abstract

Energy sustainability is a key consideration for anthropogenic activity and the development of societies, and more broadly, civilization. In this article, energy sustainability is described and examined, as are methods and technologies that can help enhance it. As a key component of sustainability, the significance and importance of energy sustainability becomes clear. Requirements to enhance energy sustainability are described, including low environmental and ecological impacts, sustainable energy resources and complementary energy carriers, high efficiencies, and various other factors. The latter are predominantly non-technical, and include living standards, societal acceptability and equity. The outcomes and results are anticipated to inform and educate about energy sustainability, to provide an impetus to greater energy sustainability.

## Introduction

Energy is utilized pervasively to provide energy services of all types. These include the provision of electricity, transportation, lighting, heating, cooling, industrial processes (e.g., refining and manufacturing) and many more. The full life cycle of energy is complex, and includes obtaining energy sources, converting them to useful forms, transporting, distributing, storing energy, and utilizing energy (Karunathilake et al. 2019). The services provided by energy allow for good living standards and support societal development.

Most countries today use energy in a manner that is not sustainable (Baleta et al. 2019). This applies to countries of all kinds (developing, industrialized, etc.) (Kumar and Majid 2020). Despite this general view, it is observed that wealthy countries appear to be using energy in a manner that is more sustainable today than before 1970. This phenomenon is illustrated in Table 1. For G7 countries, for instance, energy use per capita and real gross domestic product per capita both rose in step by about 60% between 1960 and 1973, but between 1973 and 2015, energy use per capital remained roughly constant while real gross domestic product per capita continued to rise, by roughly 100% (World Bank Group 2021). These data suggest that energy usage and GDP growth per capita became in part decoupled, implying countries can continue to generate wealth without necessarily using increasing amounts of energy through a higher energy intensity. Note that, as the data in Table 1 are just for the G7 countries, the rest of the world may not follow this behavior. G7 countries have outsourced portions of their heavy industry, which tends to be energy intensive, to developing and recently developed countries (e.g., Mexico). Hence, the net effect globally in terms of reducing energy consumption is likely less that that observed for G7 countries.

**Table 1:** Change in energy use and real gross domestic product per capita for G7 countries between 1960 and 2015

Parameter	1960–1973 (%)	1973–2015 (%)	1960–2015 (%)
Real gross domestic product per capita	+ 65	+ 95	+ 325
Energy use per capita	+ 60	− 2	+ 51

Data source: World Bank Group (2021)

Energy sustainability involves the use of energy during all aspects of its life cycle in a manner that supports the various facets of sustainable development. Energy sustainability is, therefore, a comprehensive concept that reaches beyond the use of sustainable energy resources, and can be viewed as a component of overall sustainability.

A universally accepted definition for energy sustainability does not exist, even though some definitions have been proposed (2017a; Zvolinschi et al. 2007; Chen et al. 2020a; Razmjoo et al. 2020; Suganthi 2020; Kumar and Majid 2020). A general definition can perhaps be developed by extending definitions of sustainability or sustainable development. For instance, Kutscher et al. (2019) define sustainable energy as energy produced and used in such a way that it “meets the needs of the present without compromising the ability of future generations to meet their own needs.” Grigoroudis et al. (2019) suggest that “energy sustainability is related with the provision of adequate, reliable, and affordable energy, in conformity with social and environmental requirements.” Nonetheless, defining energy sustainability is challenging due to the multidisciplinary and complex nature of energy sustainability. The present author defines energy sustainability as the provision of energy services for all people now and in the future in a manner that is sustainable, i.e., adequate to meet basic necessities, not unduly environmentally detrimental, affordable by all, and acceptable to people and their communities. Note that the author’s definition has a temporal persistence element, and that it includes communities, which adds a collective element such as can be represented by culture. Note also that the concept ‘basic necessities’ has an element of vagueness as do other aspects of definitions of energy sustainability or overall sustainability. This can be problematic, although it also provides room for interpretation by individual countries or regions. Since overall sustainability is often viewed as the simultaneous attainment of environmental, economic and societal sustainability, it is clear that energy processes affect each these facets of sustainability. This highlights the importance of energy sustainability to sustainability overall. The relevance of these ideas is increasingly in the fore, as many countries and cities are seeking to become more sustainable, and view energy sustainability as a component of this objective.

Notable environmental, economic and societal challenges are associated with energy. These need to be addressed adequately as part of achieving energy sustainability, although the process can be complex and challenging. Some of the notable challenges relate to societal inequities, excessive resource consumption, climate change and the environmental and ecological affects of other emissions, and limited energy affordability. These are made more challenging by the fact that energy prices are skewed by taxes and incentives, and political factors affect energy issues, sometimes greatly. In addition, wealth and living standards as well as population, culture and level of urbanization often vary among countries, further affecting energy sustainability. The challenges are often greater for developing and non-industrialized countries, due to lack of wealth, education, technology and many other factors. The objective of this article is to assist in addressing these challenges, by informing about energy sustainability and enhancing efforts supporting energy sustainability.

It is noted that this extends earlier work by the author, including an effort to develop a pragmatic approach to energy sustainability with relevant illustrations (Rosen 2009). The first illustration considers a thermal energy storage that receives and holds heat (or cold) until it is required, while the second assesses a heat pump that uses electricity to extract heat from a low-temperature region and to deliver it to a region of higher temperature for heating. The third illustration is cogeneration of thermal and electrical energy as well as trigeneration of electricity, heat and cold, while the final illustration considers hydrogen production based on thermochemical water decomposition driven by nuclear or solar energy.

## Energy

Energy resources are obtained from the environment. Some energy resources are renewable and some are finite in quantity and thus non-renewable. Energy systems in most countries today are principally driven by fossil fuels, but renewable energy utilization is increasing (Karunathilake et al. 2019; Hansen et al. 2019; Mehrjerdi et al. 2019; Kumar and Majid 2020). Renewable energy resources are listed with details on the main basis from which they are derived in Table 2, while non-renewable energy resources grouped by resource type are given in Table 3. Data from the IEA (2020, 2021) on global production of the energy resources are also provided for the most significant resources in terms of quality. It is seen that many types of renewable energy are derived from solar energy, including hydraulic, biomass, wind and geothermal energy (as ground energy at ground temperature) (Rosen and Koohi-Fayegh 2017). Constraints on long-term energy supplies help to determine the sustainability of the energy resources and have been discussed by Weisz (2004).

**Table 2:** Renewable energy resources

Energy resource	Main basis	Global production in 2018 (ktoe)^a^
Solar radiation (beam and scattered)	Solar energy (direct)	90,048 (includes other solar derived resources, except where listed separately)
Hydraulic	Solar energy (indirect)	362,332
Wind	Solar energy (indirect)	196,329
Wave	Solar energy (indirect)	Very small, but included under solar radiation where measured
Ocean current	Solar energy (indirect)	Very small, but included under solar radiation where measured
Ocean thermal	Solar energy (indirect)	Very small, but included under solar radiation where measured
Biomass (utilized slower than it is regenerated)	Solar energy (indirect)	1,324,214 (biofuels and waste)
Geothermal (ground source)	Solar energy (indirect)	Included in geothermal (high temperature)
Geothermal (high temperature)	Heat generated deep within the earth	2062
Tidal	Gravitational forces between earth and moon	Very small

^a^Data source: IEA (2020, 2021)

**Table 3:** Non-renewable energy resources

Energy resource type	Energy resource	Global production in 2018 (ktoe)^a^
Fossil fuels	Natural gas	3,293,124
	Oil	4,552,548
	Coal	3,893,679 (includes peat and oil shale)
	Other fossil fuels (oil sands, oil shales)	Included in coal
	Fossil fuel alternatives (oil sands, peat, wood)	Included in coal
Nuclear energy	Uranium (for fission reactors)	706,814
	Plutonium (for fission reactors)	Very small
	Other nuclear fuels (for fission reactors)	Very small
	Fusion fuels (tritium, etc.)	Very small
Biological matter-based fuels	Biomass (utilized faster than it is regenerated)	1,324,214 (biofuels and waste)
	Wastes	Included in biomass
	Peat	Included in biomass
	Wood	Included in biomass

^a^Data source: IEA (2020, 2021)

Energy carriers are the forms of energy that are utilized in processes and systems, and include fuels, electricity and heat (Rosen 2018). Some energy carriers exist in the environment while others do not and need to be produced artificially. Energy carriers, divided by energy carrier type, are listed in Table 4 for non-chemical energy carriers and in Table 5 for chemical energy carriers. Note that energy carriers do not include energy storages, which are simply temporary buffers for energy resources or carriers. Energy storages are indeed important and discussed subsequently in the article.

**Table 4:** Non-chemical energy carriers

Energy carrier type	Energy carrier
Electrical	Electricity
Mechanical	Work
Thermal (heat or cold)	Direct thermal energy
	Indirect thermal energy (via a heated or cooled substance)

**Table 5:** Chemical energy carriers

Energy carrier type	Energy carrier
Fossil fuel	Natural gas
	Petroleum
	Coal
Fossil fuel-derived	Petroleum products (gasoline, diesel, jet fuel, etc.)
	Coal products (coke, coal diesel, etc.)
Other chemicals	Substitute natural gas
	Hydrogen
	Methanol
	Ammonia

Energy is seen in Tables 2, 3, 4 and 5 to exist in various forms. Energy-conversion processes and technologies convert energy from one form to another, and can be described with thermodynamics. Of particular use are the first law of thermodynamics (the principle of conservation of energy) and the second law (the principle of non-conservation of entropy). The latter in particular helps determine energy quality and is the basis for the quantity exergy.

## Sustainability and Sustainable Development

**Fig. 1:**
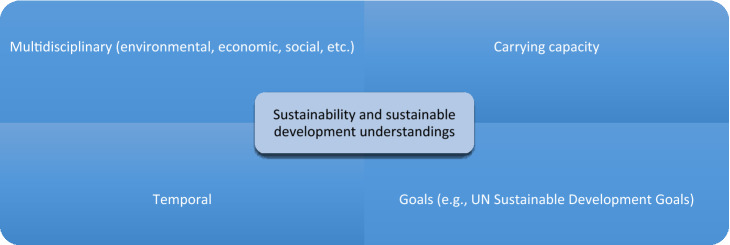
Selected understandings of sustainability and sustainable development, embodying various viewpoints

**Fig. 2:**
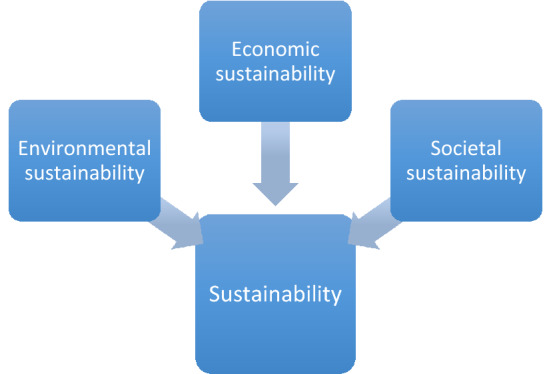
Sustainability viewed as having three principal facets: environmental, economic and societal

There are various understandings of sustainability and sustainable development, embodying various viewpoints (Rosen 2018; Baleta et al. 2019; Hengst et al. 2020; Pauliuk 2020; Dragicevic 2020; Chen et al. 2020a; Rezaie and Rosen 2020). Some of the more significant of these are illustrated in Fig. 1 and examined below (Fig. 2):

**Fig. 3:**
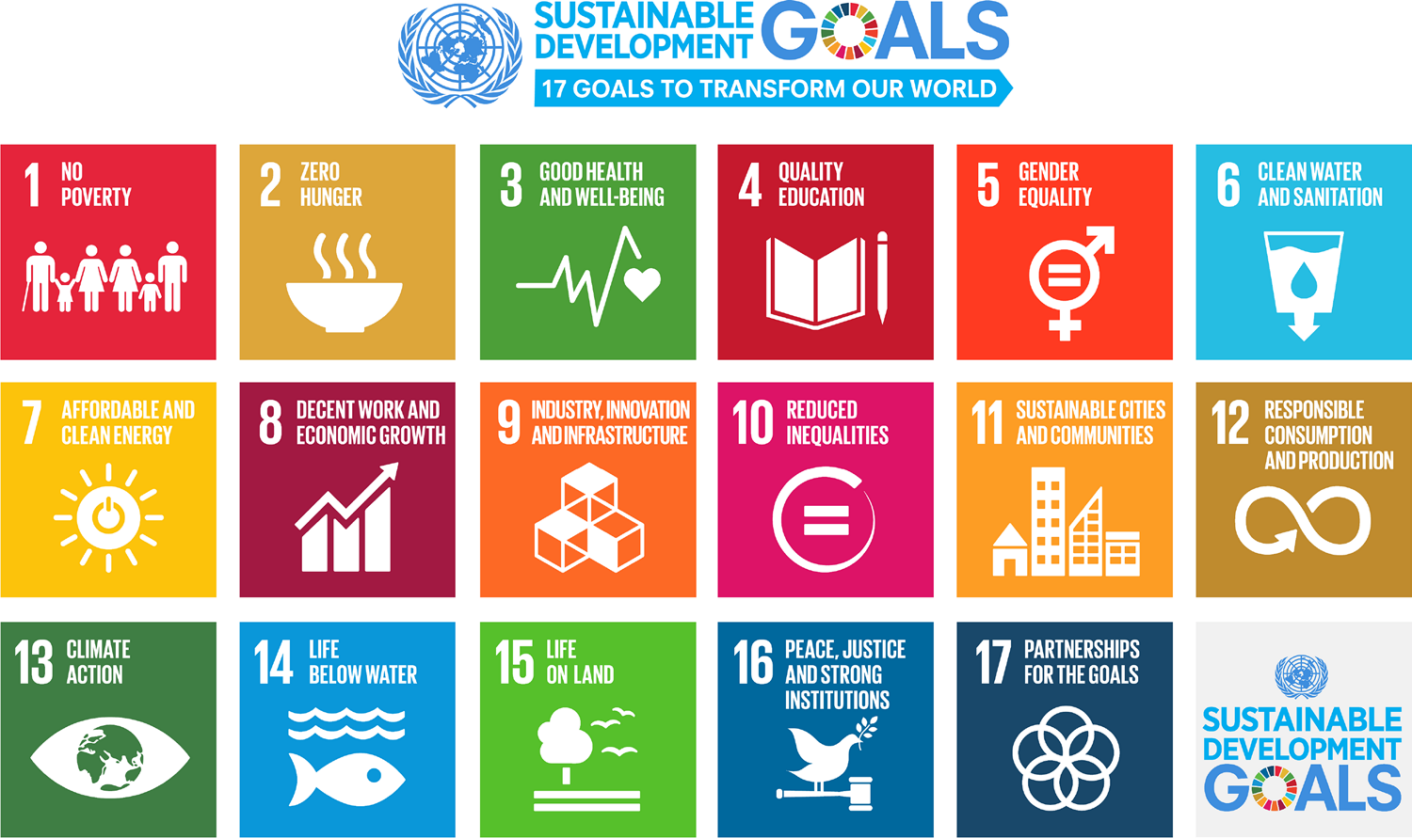
United Nations Sustainable Development Goals (SDGs) (public domain material provided by United Nations at http://www.un.org/sustainabledevelopment/news/communications-material/)

- **Multidisciplinary**. Sustainability is often viewed as multidimensional with economic, social and environmental facets (see Fig. 2). Achieving sustainability is a challenge as these three facets are often opposing, e.g., economic sustainability may necessitate sacrificing environmental sustainability, and vice versa. Jose and Ramakrishna (2021) point out the multidisciplinary nature of sustainability in their assessment of the comprehensiveness of research in the field.
- **Carrying capacity**. Sustainability can be considered in terms of carrying capacity, i.e., the maximum population supportable, given the ability of the environment to provide resources and receive wastes. This involves an environmental perspective, but is focused more on limitations. The demand and supply of resources affects carrying capacity significantly. For example, Park et al. (2020) have evaluated the carrying capacity as a measure of sustainability, for Jeju Island, South Korea.
- **Temporal**. Sustainability is usually understood as temporally lasting. The temporal scale to be considered is subjective, although a period of 50–100 years is fairly often viewed as reasonable for many sustainability considerations (Graedel and Allenby 2010). Yet, this time frame can be disputed, especially for energy issues that can straddle centuries or more. For example, the lifetimes in terms of reserve base for fossil fuels have been estimated to be 51 years for oil, 53 years for natural gas and 114 years for coal, based on annual consumption rates (BP 2016). Thus coal-burning could be viewed as sustainable for the next 100 years or so based on the available resources, but then they would be practically exhausted clearly making them coal use not sustainable (and that is not considering the pollution and climate change effects from coal combustion). This contrasts with solar and wind energy, which have no date to exhaustion (until the sun ‘dies’ through running out of hydrogen, in about 5 billion years). Clearly, too short a period for evaluating sustainability is not helpful since most activities are sustainable for years, but too long a period is intractable.
- **Goals**. Sustainability can be described in terms of aims or goals. Notable advances have been made in this approach (Rosen 2017c) with the adoption of the UN Sustainable Development Goals for 2015–2030, which encompass 17 broad goals (see Fig. 3) (United Nations 2015). Adopted at the 70th Session of the United Nations General Assembly in 2015, the UN Sustainable Development Goals form part of the 2030 Agenda for Sustainable Development. It is noted that work by the United Nations on sustainability has a lengthy history, extending back to the World Commission on Environment and Development (1987) and its 1987 report ‘Our Common Future,’ which defined sustainable development as ‘development that meets the needs of the present without compromising the ability of future generations to meet their own needs.’

## Sustainability and Energy

**Fig. 4:**
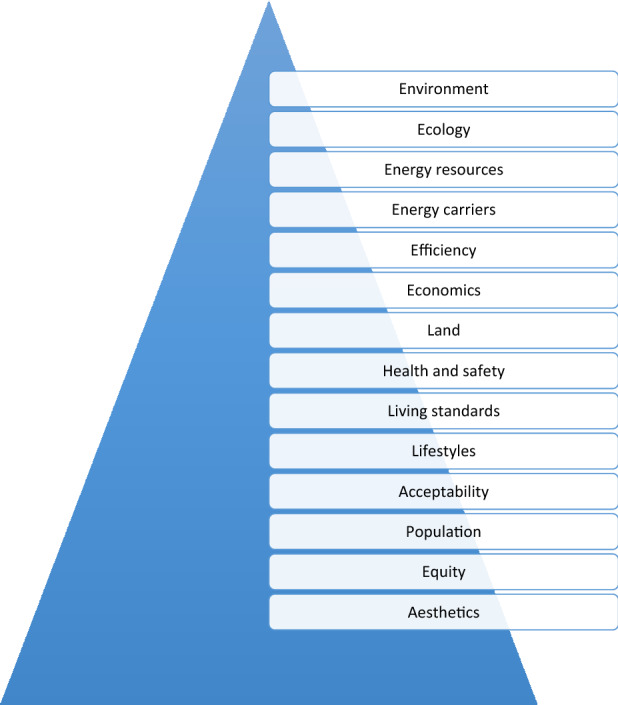
Principal issues for achieving or shifting towards energy sustainability

Based on the present author’s definition of energy sustainability cited earlier (the provision of energy services for all people now and in the future in a manner that is adequate to meet basic necessities, not unduly environmentally detrimental, affordable by all, and acceptable to people and their communities), it is evident that various issues impact how energy resources can be sustainable. Many of these issues are illustrated in Fig. 4. Through these issues, key needs for energy sustainability can be developed. These are listed in Table 6 along with interpretations of them.

**Table 6:** Principal requirements for energy sustainability

Area	Requirement	Interpretations
Environment and ecology	Low environmental and ecological impacts	Mitigate the environmental and ecological impacts of processes and systems over their life cycles in a sustainable manner
Energy resources and carriers	Sustainable energy resources and complementary energy carriers	Extract and harvest energy resources that are sustainable, and utilize complementary energy carriers that are supportive of all facets of energy sustainability
Efficiency	High efficiencies	Raise efficiencies of processes and systems in a sustainable manner
Economics	Economic sustainability and affordability	Attain economic sustainability and ensure affordability of sustainability measures for all people
Others	Tackle other, mainly non-technical, aspects of energy sustainability	Address requirements for health and safety
		Manage resource and land use appropriately
		Create good living standards and desirable lifestyles
		Ensure societal acceptability of energy sustainability measures and involvement in developing them
		Manage demands and expectations for increased energy services and utilization
		Account for increasing population
		Support equity and fairness, including on a temporal basis (e.g., for future generations) and spatial basis (e.g., among countries)
		Satisfy desires for good aesthetics and cleanliness

The key needs for energy sustainability are examined in the remainder of this section.

### Low Environmental and Ecological Impacts

Numerous environmental and ecological impacts are associated with energy systems over their lifetimes, ranging from local to national and international. Energy-related environmental and ecological impacts must be adequately addressed to attain energy sustainability, as their mitigation supports energy sustainability (Rosen 2012, 2018; Sciubba 2019; Veiga and Romanelli 2020).

Some of the more notable environmental and ecological impacts linked to energy are as follows:

- Global climate change due to greenhouse gas emissions (Almazroui et al. 2019; Scott 2007).
- Abiotic resource depletion, due to the excessive use of non-biological and non-renewable raw materials (Graedel and Allenby 2010).
- Acid precipitation and acidification due to emissions of substances such as sulfur dioxide and nitrogen oxides (Rosen 2012).
- Stratospheric ozone depletion, which allows increased levels of ultraviolet radiation to reach the surface of the earth, causing adverse health effects (Razmjoo et al. 2020).
- Ecotoxicity and radiological exposures, and the health problems they can cause, such as those due to radioactivity in building materials (Pillai et al. 2017).

Climate change, as a consequence of global warming, is caused mainly by emissions of greenhouse gases (especially carbon dioxide), and is particularly concerning due to its potentially severe consequences (loss of land fertility in near equatorial regions, rising ocean levels and flooding of many cities, more frequent and stronger storms, etc.). These effects and others have recently been quantitatively assessed (Chen et al. 2020b). By disrupting the earth–sun–space energy balance, these emissions lead to increases in mean global temperatures and consequential changes in climates. Low-carbon and carbon-free energy options are needed for climate change mitigation, as they can significantly lower emissions of the primary greenhouse gas, carbon dioxide, which is emitted through carbon fuel combustion.

Many effects of climate change have been studied, such as its impacts on hydro-meteorological variables and water resources (Almazroui and Şen 2020) and on water engineering structures (Almazroui et al. 2019). In addition, responses to climate change in the form of mitigation efforts have been examined, including carbon sequestration (Were et al. 2019) and carbon emission reduction (Khalil et al. 2019). Many of the effects and responses mentioned here relate to energy use, directly or indirectly.

For comprehensive and meaningful assessments of environmental and ecological impact, the overall life cycle of an energy system or activity needs to be considered, starting with the harvesting and processing of energy and other resources, and on to their utilization and ultimate disposal. Life cycle assessment (LCA) is an effective methodology for analyses (Graedel and Allenby 2010). LCA has been applied extensively to a broad range of activities (Ben-Alon et al. 2019; Lodato et al. 2020; Lu and Halog 2020), including energy processes (Sadeghi et al. 2020; Mendecka et al. 2020) and communities (Karunathilake et al. 2019).

### Sustainable Energy Resources and Complementary Energy Carriers

Sustainable energy resources are crucial to energy sustainability, as are complementary energy carriers that allow those energy resources to be exploited or facilitate sustainable energy options. On the one hand, fossil fuels (see Table 3), the most common non-renewable energy resources, are finite in nature. On the other hand, renewable energy sources, including solar, hydraulic, wind, biomass, and geothermal energy (see Table 2), can be sustained for extremely long. Renewable energy resources also mitigate greatly or avoid greenhouse gas emissions, among other advantages. Some special cases are worth noting:

- Uranium (nuclear energy fuel) is a non-renewable energy resource but it does not contribute significantly to climate change, and the lifetimes of nuclear fuel assuming their use in advanced breeder reactors is thought to exceed 1000 years, so it is often viewed as a sustainable energy option (Al-Zareer et al. 2020a). For example, Fetter (2009) estimated the extraction of uranium from seawater would make available 4.5 billion metric tons of uranium, representing a 60,000-year supply at present usage rates, while fuel-recycling fast-breeder reactors could match today’s nuclear output for 30,000 years, based on data of the Nuclear Energy Association (NEA). But this is contentious, as these very long nuclear fuel lifetimes remain hypothetical, while current actual nuclear power plants consume uranium at a much faster rate relative to reserves, in the process generating significant amounts of waste with half-lives that are significantly longer than 1000 years. The supply was estimated at 230 years in 2009 (Fetter 2009), based on identified uranium resources of total 5.5 million metric tons and an additional 10.5 million metric tons still undiscovered and the consumption rate at that time. Moreover, only very few nuclear plants are “fast breeder reactors”.
- Biomass may or may not be considered a renewable energy option, depending on its rates of utilization and replenishment. Regardless of the classification, decisions on using various types of biomass depend on both their costs (in terms of energy use quantity and rate, net quantity of carbon used, economics), and their benefits (net quantity of carbon emissions avoided, financial savings, etc.). The potential for biomass use to be sustainable often includes energy return on investment (EROI), which is the ratio of the amount of usable energy delivered from a particular energy resource to the amount of energy used to obtain that energy resource (Hall et al. 2014; Wang et al. 2021). This value has ranged from 0.64 (below the breakeven value of 1) for early biomass uses for producing ethanol to as high as 48 for some particular processes involving molasses, and typical values today are 4–5. Biomass is generally not sustainable when EROI values are near or below 1. In addition, it is noted that biomass typically has a low-energy conversion efficiency (relative to values for fossil fuels) and its production sometimes displaces food production, reducing in those cases its prospects as a sustainable energy resource.
- Wastes, which can include some forms of biomass, are sometimes viewed as a renewable energy resource and sometimes are not, given people can modify behaviors to reduce wastes greatly.

Much research on energy resources has been reported, including electricity generation from food waste through anaerobic digestion (Ali et al. 2019; Rezaie and Rosen 2020) and hydroelectric generation (Udayakumara and Gunawardena 2018), and solar energy applications (Hachem-Vermette et al. 2019; Sun et al. 2019). These studies collectively demonstrate the importance of energy sources in discussions of sustainability, and illustrate the feasibility of such technologies in practical applications.

Energy carriers, which include electricity, thermal energy and secondary fuels (see Tables 4, 5), play an important although less prominent role in energy sustainability. Before they can be utilized, energy resources often require conversion to other energy forms or carriers, e.g., solar photovoltaic panels to produce electricity for renewable energy resources, petroleum refineries for non-renewable energy resources, and hydrogen production from both types of energy resources. The latter example supports the idea of a hydrogen economy, in which hydrogen and electricity are the two main energy carriers (Scott 2007; Rosen 2017b; Gnanapragasam and Rosen 2017; Moharamian et al. 2019; Abe et al. 2019; Endo et al. 2019; Fonseca et al. 2019; Chapman et al. 2020; Al-Zareer et al. 2020a; Mehrjerdi et al. 2019**)**. Energy sustainability is supported well by this combination of energy carriers since most chemical energy needs can be satisfied by hydrogen (and select hydrogen-derived fuels) and non-chemical energy needs by electricity.

### High Efficiencies

High efficiency in a holistic sense is broad, covering:

- high device and system efficiencies,
- energy conservation,
- energy management and matching of energy demands and supplies,
- appropriate utilization of energy quality, and
- advantageous fuel substitution.

This holistic sense is adopted here. High efficiency supports energy sustainability by expanding the benefits of energy technologies, whether renewable or not, although the benefits are more pronounced for non-renewable energy resources. High efficiency elongates the lives of finite-energy resources and lowers the capacities needed for energy devices. High efficiencies often can improve societal metrics such as standard of living, quality of life, and satisfaction. For instance, the US and Sweden have similar gross domestic products (per capita), but the latter exceeds the US in most social indicators and utilizes 40% less energy (per capita) through more efficient buildings, smaller automobiles and better public transit, and higher gasoline taxes (Rosen 2018). Advanced methods are available to help attain high efficiencies, e.g., exergy analysis provides insights not available via conventional energy methods (Rosen 2012; Dincer and Rosen 2021b, 2015) and has been applied widely (Morosuk and Tsatsaronis 2019; Sciubba 2019; Veiga and Romanelli 2020; Kumar et al. 2020).

### Economic Sustainability and Affordability

Energy sustainability necessitates that the energy services required for basic needs be economically affordable by most if not all people and societies (Rosen 2011). However, the economics of energy sustainability measures usually need to be reasonably competitive with conventional approaches to find acceptance and adoption, although it is noted that some efficiency measures, like some environmental impact mitigation measures, can over time sometimes pay for themselves or save money. Government incentives also can enhance affordability.

Of course, many other factors affect economic sustainability and affordability. First, the economic “externalities” associated with fossil fuel combustion, i.e., the environmental costs that are not accounted for in the cost of production, are normally not counted. When externalities are properly accounted for, the economics improve for non-polluting energy forms such as wind and solar, and can become more favorable than the economics for fossil fuels. For example, Bielecki et al. (2020) show that the costs of externalities for fossil fuels and peat are typically 10–100 times greater than those for sustainable energy forms such as hydraulic, solar, wind, biomass and nuclear. Furthermore, economies of scale are an important factor in lowering economic costs, thereby making energy sources more sustainable. In addition, the economics of energy often fluctuates in response to energy resource scarcity or abundance, political instability for the case of finite-energy resources such as oil, natural gas, and uranium. Finally, the intermittency of some renewable energy forms such as wind and solar can raise their costs.

### Others

**Fig. 5:**
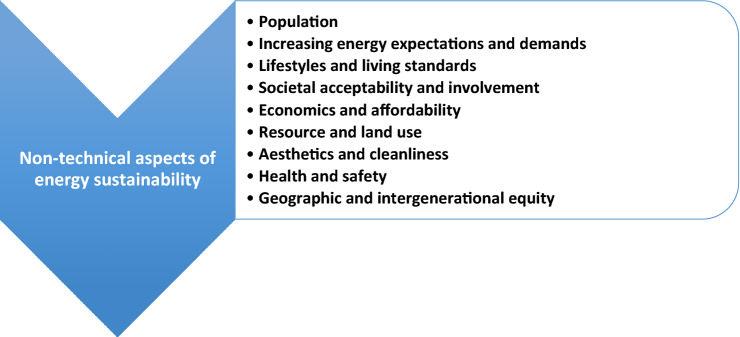
Selected non-technical aspects of energy sustainability

Other needs exist for energy sustainability and need to be addressed, and a great number of these are non-technical. Selected needs are shown in Fig. 5 and discussed below:

- **Geographic and intergenerational equity.** For energy sustainability, equity is needed among present and future generations and among developed and developing countries in terms of energy access. Being concerned about future generations is important to the temporal aspect of sustainability, and involves considering the responsibility of people to consider the effects of their actions today, and their motivations, on any harm that may be brought to future generations. This involves trade offs. Concerns about energy access developed and developing countries raises issues of fairness and other trade offs, e.g., is it reasonable for countries that became wealthy in large part through extensive use of fossil fuels to ask developing and poorer nations to forego the use of fossil fuels and to use more sustainable energy forms, even if the costs are higher.
- **Increasing population, energy demands and living standards.** Increasing global population must be accounted for in energy sustainability measures and strategies, as it places stresses on the carrying capacity of the planet and the environment. Furthermore, the rising demand and desire for energy resources with increasing wealth, especially as developing countries attain higher living standards, also makes energy sustainability more challenging. Energy sustainability can be assisted by measures involving transformations in lifestyles and reductions in energy demands, although this is usually very challenging in general and especially for policy makers. Behavioral modification requires recognition that present development trends are unsustainable over time. Many of these issues have been studied previously, such as the vulnerability of livelihoods in regions and countries (Qaisrani et al. 2018).
- **Resource and land use.** Balances are often necessary to preserve resources and land for the uses for which they are most needed. For instance, land uses for growing biomass for biofuels needs to be appropriately weighed against agriculture needs, flooding large tracts of land needs to be balanced against hydroelectric generation requirements, and ecosystem preservation needs to be balanced against long-distance electrical transmission corridors.
- **Societal acceptability and involvement.** For acceptance of energy sustainability measures, societies and their populations must be informed, involved in decisions, and supportive of them. This normally necessitates thorough consultation, and is particularly important when special or disadvantaged communities are involved, such as some indigenous communities.
- **Aesthetics and cleanliness.** Energy sustainability measures should not degrade unduly the aesthetic appeal and cleanliness of the environment, for societal and other reasons. Even renewable energy resources can be aesthetically problematic, e.g., large solar PV installations and wind farms. Of course, aesthetics are a personal matter and vary from one person to another, sometimes considerably, often making it challenging to find the appropriate trade off.
- **Health and safety.** In strategies and plans for energy sustainability, energy options must be healthy and safe, as evidenced by concerns associated with the COVID-19 pandemic that began in 2019. This issue has spawned much research, e.g., an investigation of the impact of daily weather on the temporal pattern of COVID-19 outbreaks (Gupta et al. 2020).

Note that these non-technical factors of energy sustainability are at times interconnected, related and overlapping. Note also that many of the non-technical factors are often addressed if the technical factors discussed previously are addressed suitably. An example: factors such as public acceptability, economics, and equity need to be accounted for when choosing among sustainable energy options. Examining these issues makes it apparent that energy sustainability is politically sensitive, due to the political nature of many of the issues raised in the above points. Even though these points may be recognized already, they are included here for completeness, especially in light of their importance.

## Methods for Enhancing Energy Sustainability

**Fig. 6:**
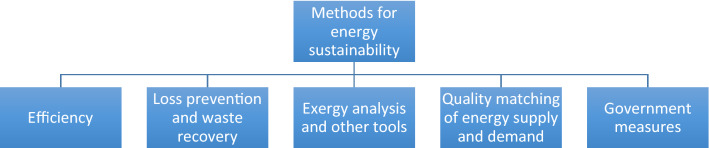
Selected methods for enhancing energy sustainability

A selection of energy methods that can help enhance energy sustainability directly or indirectly, shown in Fig. 6, are now described.

Efficiency, loss prevention and waste recovery can all help enhance energy sustainability. Appropriately high-efficiency devices and systems facilitate and contribute to energy sustainability, e.g., heaters, chillers and air conditioners, pumps and compressors, motors and fans, and lighting have higher efficiencies today than in the past, the latter due to more efficient bulbs, lower lighting intensities, task lighting, and lighting occupancy sensors. Efficiency can also be improved by preventing losses, e.g., with better insulation, and by recovering energy wastes, e.g., by waste heat recovery.

Exergy analysis and other advanced tools can support energy sustainability. Thermodynamic performance can be better assessed, improved and optimized with exergy analysis rather than energy analysis, since the former evaluates more meaningful efficiencies and better pinpoints inefficiencies. Based on exergy, a measure of energy usefulness or quality or value (Dincer and Rosen 2021b), exergy methods have been applied increasingly in recent years (Dincer et al. 2017; Moharamian et al. 2019). In addition, quality matching of energy supply and demand can also support energy efficiency. It is usually more efficient to supply an energy quality better matched to energy demand instead of supplying an exceedingly high-quality energy form, and thus having a quality mismatch, a result well illustrated with exergy analysis. For example, supplying heating for aquaculture at 20 °C with a natural gas combustor capable of heating to 1000 °C is mismatched compared to using simple solar thermal collectors operating at 40 °C.

Governments can also apply incentives (technically and/or societally directed) and enforcement activities to support energy measures. These can be mandatory or voluntary, depending on circumstances and needs. Modifications to lifestyles and societal structures can also reduce energy use, e.g., shifting North America’s transportation preference to mass transit from automotive, in part by changing energy taxation and environmental restrictions.

## Technologies for Enhancing Energy Sustainability

**Fig. 7:**
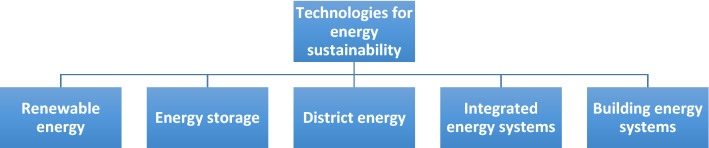
Selected technologies for enhancing energy sustainability

Sample energy technologies that can help enhance energy sustainability directly or indirectly, shown in Fig. 7, are now described. Note that the methods discussed in the prior section are intended to include techniques and approaches for improving energy sustainability, while the technologies covered in this section focus on specific technologies that can be employed to improve energy sustainability. Of course the methods can be applied to technologies, but the focus of the prior section was on methods and techniques.

Utilizing renewable energy sources (e.g., hydraulic, solar, wind, geothermal, biomass, wave, tidal and ocean thermal energy) can contribute to energy sustainability, as they can be sustained for long time periods and have low environmental emissions and impacts. These sources have been extensively investigated, e.g., the amplitudes and phases of tides near power stations (Madah 2020) as well as the impacts of potential sea-level rise on tides (Lafta et al. 2020). Energy storage can also support energy sustainability, in part by offsetting the intermittency of some renewable energy resources (Krishan and Suhag 2019). Energy storage can also store energy until it is economic to deploy, and enhance efficiency and energy management (Al-Zareer et al. 2020b). There are various types of energy storage (Koohi-Fayegh and Rosen 2020), including thermal energy storage (Dincer and Rosen 2021a), underground storage using borehole heat exchangers (Sliwa et al. 2019) and batteries (Al-Zareer et al. 2020b). Energy storage is increasingly being employed in building and HVAC systems (Dincer and Rosen 2015), and in renewable energy systems involving hybrid energy schemes (Rekioua 2020) and microgrids (Al-Ghussain et al. 2020).

Integrated energy systems, based on renewable and/or non-renewable energy technologies, can enhance energy sustainability and efficiency, e.g., polygeneration systems (Rosen and Koohi-Fayegh 2016; Calise et al. 2019; Rokni 2020; Mendecka et al. 2020; Kasaeian et al. 2020), and linking separate systems advantageously such as in cascading energy systems (Campana et al. 2019; Liu et al. 2020; Rokni 2020).

Building energy systems can be modified to enhance energy sustainability, e.g., using active systems such as renewable energy resources and passive technologies such as Trombe walls, multiple glazing windows and selective window coatings, daylight harvesting, insulation, weatherstripping and caulking. Note that behavior, culture and lifestyle also can affect the success of energy efficiency measures in buildings, as was illustrated for China (Zhang and Wang 2013). Energy sustainability can also be enhanced via district energy systems, in which thermal energy can be generated in heating or cooling facilities, using renewable energy or conventional resources, and transported to users. District energy systems are used in many cities and traverse a wide range of distances (Rosen and Koohi-Fayegh 2016). Buildings in many cities are connected through district energy systems that provide space and water heating and space cooling.

## Illustration

**Fig. 8:**
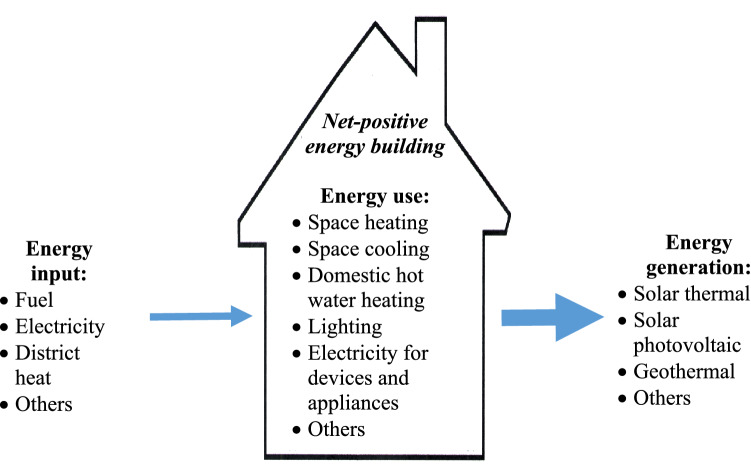
Net-positive energy building, in which energy generation from renewable energy resources exceeds energy use over a typical year

**Fig. 9:**
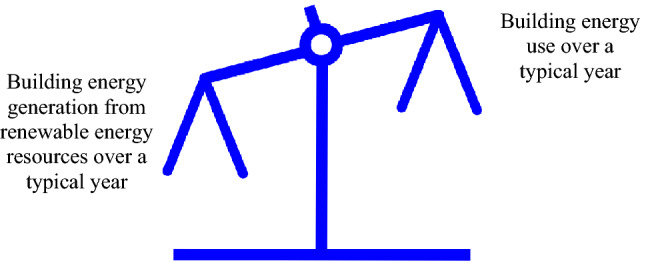
Imbalance of a net-positive energy building, highlighting how energy use over a typical year is less than energy generation from renewable energy resources

In this illustration, we consider net-positive energy buildings. A net-positive energy building over an average year generates more energy from renewable energy sources than it uses, as shown in Fig. 8, and can support energy sustainability (Rosen 2015; Endo et al. 2019; Delavar and Sahebi 2020; Tumminia et al. 2020; Singh and Das 2020). A net-positive energy building uses energy for a variety of tasks and generates energy from various renewable energy resources, and achieves net-positive energy status through advanced design and exploitation of technologies such as advanced automation, controls, component integration, energy storage, lighting and HVAC. One of the main upcoming “other energy uses” for electricity in Fig. 8 will likely be for vehicle energy (e.g., for electric automobiles). Such utilization of energy is likely to prove both cost effective and environmentally friendly. A net-positive energy building generates more electrical plus thermal energy from renewable energy sources than it uses over an average year, as shown in Fig. 9. Such buildings are net energy generators, rather than net energy users, like most buildings today. Research on net-zero and net-positive energy buildings has been reported (Athienitis and O’Brien 2015; Mehrjerdi et al. 2019; Sun et al. 2019), while the International Energy Agency included an annex on “Towards Net-zero Energy Solar Building” and Canada launched in 2011 the Smart Net-zero Energy Buildings Strategic Research Network (http://www.solarbuildings.ca). The net-zero and net-positive energy building concepts can be expanded to include transportation devices that are part of the building (Garmsiri et al. 2016; Sun et al. 2019) and to net-zero and net-positive energy communities (Rad et al. 2017; Hachem-Vermette et al. 2019; Karunathilake et al. 2019; Nematchoua et al. 2021).

As a numerical example that correlates with the qualitative presentation in Fig. 8, a performance assessment by Zomer et al. (2020) of PV systems installed at a positive energy building is considered here. The building is the Fotovoltaica/UFSC solar energy laboratory (http://www.fotovoltaica.ufsc.br) in Florianópolis, Brazil (27° S, 48° W). Although originally designed as a zero-energy building with PV systems on rooftops and façades, additional PV systems were installed on the same location on a carport and an electric bus (eBus) shelter and charging station, and ground-mount PV systems with single-axis solar tracking installed. The system then had a peak PV generation capacity of 111 kW. Energy generation and consumption were analyzed on monthly bases, and the key results are listed in Table 7. The total PV generation in the period could supply 97% of the building (including eBus) energy consumption, accounting for actual performance and downtime for R&D activities. In that case, the building was almost a net-zero energy building (for which the energy supply would meet 100% of the consumption). However, when the systems operate all the time at their optimal performance, the PV system can supply 134% of the building (including eBus) energy consumption, making it a positive energy building.

**Table 7:** Energy generation and consumption for Fotovoltaica/UFSC solar energy laboratory

System operation mode	Ratio of energy generation from PV system to building energy need (including eBus)	Status regarding net energy use
Actual over test period, including downtime events (for research activities)	0.97	Near net-zero energy building
Optimal performance all the time	1.34	Positive energy building

## Conclusions

Energy sustainability is described, with a focus on environmental perspectives, as are methods and technologies to enhance it. In essence being a key component of sustainability, the significance and importance of energy sustainability becomes clear. Requirements to increase energy sustainability are discussed, including low environmental and ecological impacts, sustainable energy resources and complementary energy carriers, high efficiencies, low environmental impacts, and various other predominantly non-technical factors. The latter include living standards, societal acceptability and equity. Interrelations among these are examined. Examples and illustrations are described that help to indicate the benefits of enhancing energy sustainability. The illustrations also indicate the complexity of energy sustainability and the factors that contribute to it, showing how challenging it can be to enhance energy sustainability. Net-positive energy buildings in particular illustrate the benefits and challenges. The outcomes and results serve to inform and educate about energy sustainability, to provide an impetus to move people in particular and civilization in general towards it.
